# Obtaining Electrospun Membranes of Chitosan/PVA and TiO_2_ as a Solid Polymer Electrolyte with Potential Application in Ion Exchange Membranes

**DOI:** 10.3390/membranes13110862

**Published:** 2023-10-27

**Authors:** Elio Enrique Ruiz Gómez, Jose Herminsul Mina Hernandez

**Affiliations:** 1 Grupo de Investigación en Estudios Aeroespaciales—GIEA, Escuela Militar de Aviación Marco Fidel Suárez (EMAVI)–Fuerza Aeroespacial Colombiana, Carrera 8 No. 58-67, Cali 760011, Colombia; elio.ruiz@emavi.edu.co; 2 Grupo de Investigación Materiales Compuestos—GMC, Universidad del Valle, Calle 13 No. 100-00, Cali 760042, Colombia

**Keywords:** solid polymeric electrolyte, titanium oxide, binary blends, electrospinning, fuel cells

## Abstract

A binary polymeric blend was prepared using chitosan (CS) and polyvinyl alcohol (PVA) at a ratio of 80:20, respectively, to obtain a solid polymeric electrolyte with possible application for the generation of an electric current in proton or anion exchange electrochemical cells. With a 6% *m*/*m* solution, a membrane was formed using the electrospinning technique, and the influence of the incorporation of titanium oxide (TiO_2_) nanoparticles, at a concentration between 1000 and 50,000 ppm, on the physicochemical properties of the material was evaluated. The micrographs obtained by SEM revealed that the diameter of the nanofibers was close to 100 nm. Likewise, it was found that the incorporation of the nanoparticles affected the moisture absorption of the material, reaching a predominantly hydrophobic behavior in the composite with the highest concentrations of these (2% absorption), while for the lowest content of the filler, the absorption reached values close to 13%. On the other hand, Thermogravimetric Analysis (TGA) showed lower dehydration in the fibrous composite with a 1000 ppm TiO_2_ content, while Differential Scanning Calorimetry (DSC) showed that these nanoparticles did not significantly affect the thermal transition (Tm) of the composite. Additionally, with the incorporation of nanoparticles, a shift in the Tg from 44 to 37 °C was found concerning the unfilled binary membrane, which increased the possibility of achieving higher ionic conductivities with the nanocomposites at room temperature. Complex Impedance Spectroscopy determined the material’s activation energy, decreasing this by adding the TiO_2_ filler at a concentration of 1000 ppm. On the other hand, when the membranes were doped with a 1 M KOH solution, the fibrous structure of the membrane changed to a porous cork-like configuration. In future research, the electrospun membrane could be used in the development of a composite to validate the energy efficiency of the new solid polymer electrolyte.

## 1. Introduction 

In sustainable development, research on alternative energy sources has sought to regulate the environmental impacts associated with energy demands (Atapour et al., 2020) [1]. The high energy efficiencies of electrochemical cells compared to petroleum-based fuel machines have offered a more environmentally friendly alternative. The polymer electrolyte membrane (PEM) is an important part of electricity generation in a direct methanol alkaline fuel cell (DMAFC) or proton exchange membrane fuel cell (PEMFC). Tripathi and Shahi (2020) [2] highlighted three aspects of the development of PEM: the good mechanical behavior of the membrane, the effect of the ceramic filler on the polymer matrix in balancing its hydrophilic nature, and the chemical activation (doping) of the membranes to produce PEM with high efficiencies and low costs. In this sense, Ruiz et al. (2020) [3] reported low water losses (7% or less) with temperature increases up to 140 °C in a binary polymeric membrane produced by PVA/CS casting; this phenomenon was due to the synergistic effect of the incorporation of the nanofiller (1000 ppm TiO_2_). 

One of the aspects mentioned by Ayse and Bozkurt (2012) [4] to achieve the optimal performance of a PEM is the proper hydration of the ionomer structure of the polymer. In this regard, Wang and Wang (2013) [5] argued that water retention was favored by increasing the temperature with the proper dispersion of the inorganic filler in the membrane. The authors mentioned the behavior of chitosan (CS) as a low-cost and environmentally friendly biopolymer with potential use as an electrolyte.

Polyvinyl alcohol (PVA) is a biodegradable synthetic polymer that presents high ionic conductivities when the polymeric matrix has been do-doped with hypophosphorous acid, as widely evidenced in different studies [6,7,8,9,10,11]. Quintana et al. (2019) [11] mentioned that blending chitosan with synthetic polymers, such as PVA, is a convenient method for preparing synthetic biodegradable polymers with versatile properties, such as good water absorbance and improved mechanical properties. Additionally, Gonçalves et al. (2017) [10] found that increasing the CS content contributed significantly to the higher toughness and lower brittleness of the electrospun membrane when blended in a PVA/CS composition from 70:30 to 85:15, also commenting on excess use of the in-corporation of PVA limits due to its high solubility in water. Yong et al. (2007) [12] reported strong hydrogen bonding interactions between PVA and CS molecules. The same authors found that crystalline microstructures of nanofibers did not develop well due to the rapid solidification of electro-spun fibers. Nanocomposites filled with nanoparticles allow for a reduction in the glass transition temperature Tg and an increase in the ionic conductivity at room temperature. Over the last decade, there have been numerous research efforts and new methodologies for the development of materials with a solid polymer electrolyte (SPE) nature described by Xiao et al. (2021) [13] and Habiba et al. (2016) [14]. In this regard, Yang et al. (2018) [15] presented a composite (PVA/CS) formed with the combination of casting and electrospinning techniques as a potential candidate solid polymeric electrolyte for direct methanol fuel cell applications with desirable electrical properties and methanol permeability. 

Consequently, binary membranes in the PVA:CS ratio of 80:20 were selected for the study in this work. TiO_2_ nanoparticles were dispersed in the binary matrix to produce a solid plasticizing effect and improve the physicochemical properties of the membranes produced by the electrospinning technique. To study the effect of the chemical activation of the nanofibers, they were subjected to a doping process by very briefly immersing them in a solution with potassium hydroxide (1 M KOH).

## 2. Materials and Methods

### 2.1. Materials

Polyvinyl alcohol (PVA) with a molecular weight (Mw) between 85,000 and 124,000 g/mol (99% of hydrolysis degree) was used. The Chitosan (CS) used had an Mw with values between 50,000 and 124,000 g/mol and a degree of deacetylation between 75 and 85%. The titanium oxide (TiO_2_) nanoparticles belonged to the anatase phase with an average particle size of 25 nm. The acetic acid used had a purity of 99%, while the potassium hydroxide KOH used was 90%. All reagents used in the investigation were purchased from Sigma Aldrich and were used as received without further purification.

### 2.2. Preparation of Polymeric Nanofibers PVA/CS-x TiO_2_

**Figure 1:**
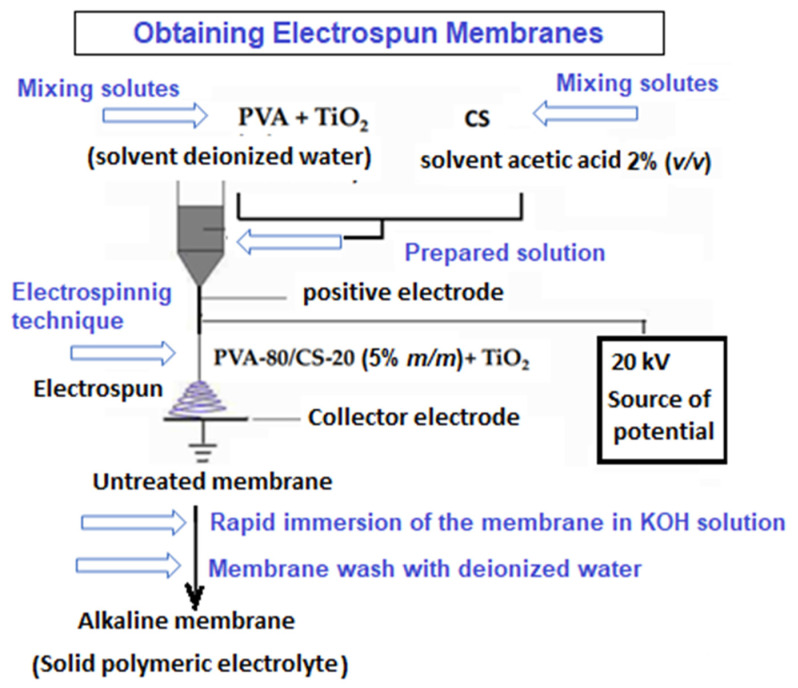
Diagram of the obtaining electrospun membranes. Abbreviations: PVA, polyvinyl alcohol. CS, Chitosan.

For the preparation of the polymeric nanofibers with a PVA/CS ratio of 80/20 (mass/mass%) and TiO_2_ nanoparticles in a concentration range between x = 1000 and 50,000 ppm of the total solute, a solution was prepared with a 6% concentration, as previously described by Ruiz et al., 2020 [3]. Finally, membranes with nanofibers and an average diameter of 100 nm were formed through the electrospinning technique, following the same method reported in similar studies by Chun et al., 2008 [16]. The polymer solution was deposited in a 10 mL syringe provided at its end with a metal capillary (diameter approximately 0.3 mm) and was subjected to electric potential for 4 h at room temperature (25 °C). A high-voltage source (0 to 30 kV) generated the electric field. The positive terminal of the source was connected to the metal capillary at the outlet of the solution and the negative terminal to a flat electrode of a circular shape, with a gold coating used as a collector; this, in turn, was connected to the tie-rod. The conditions in the electrospinning process at room temperature included a voltage of 21 kV, a distance between electrodes of 15 cm, and a solution pumping flow rate of 0.008 µL/h. Under these parameters, the fibrous composite called PVA/CS-x TiO_2_ was obtained, forming nanofibers with nanofiller concentrations within the binary mixture of 1000, 5000, 10,000, and 50,000 ppm TiO_2_. The fibrous membranes were placed in a chamber with silica as desiccant material at an ambient temperature for 24 h. Then, the membranes were immersed in 1 and 2 M KOH solutions for a 60 s period to perform the doping process. Then, the material was removed from the alkaline solution, and its surface was washed with distilled water. Finally, the membranes were dried in an oven at 37 °C for 24 h. Figure 1 shows schematically the procedure that was followed to obtain the alkaline membranes.

### 2.3. Morphological Characterization

A JSM 6490 LV (Tokyo, Japan) model JEOL scanning electron microscope was used to characterize the fibrous membranes’ surface section and determine their morphology. The electro-spun sample collected on an electrode (aluminum foil) was coated on its free surface with a gold film using Denton Vacuum Model Desk IV thin film deposition equipment to generate a conductive surface area. The images were obtained in the backscattered electron mode using an accelerating voltage of 20 kV.

### 2.4. Thermal Characterization (DSC and TGA)

Electro-spun samples with a mass of approximately 5 mg were used and subjected to a heating rate of 10 °C/min and a temperature range from 0 to 400 °C. A TA model Q100 DSC Differential Scanning Calorimeter (New Castle, DE, USA) was used to determine the thermal transitions of the membranes (Tm and Tg). The degree of crystallinity (*X_C_*) in the membranes was estimated using the model presented in Equation (1).

(1) $$X_{C}(\%)=\left(\;\frac{{∆H}_{f}}{w_{pVA}\times {∆H}_{f}^{0}}\right)\times 100$$

where ${∆H}_{f}^{0}$ is the enthalpy of fusion for 100% crystalline PVA, with a theoretical value of 138.6 J/g as reported by Guan et al. (2017) [17]; ${∆H}_{f}$ is the enthalpy of fusion in the sample; and $w_{pVA}$ is the mass fraction of PVA used in the binary blend. TGA measurements were performed on a model 2050 TA thermogravimetric analysis equipment using a heating rate of 10 °C/min, a sample mass of approximately 5 mg, and a protective atmosphere with a constant flow of gaseous N_2_.

### 2.5. Moisture Absor Ption

The polymeric membranes previously prepared by electrospinning were placed for 12 h in a chamber and were provided with a silica desiccant, reaching an equilibrium relative humidity (RH) of 7%. The mass of the dehydrated membranes was recorded as $m_{1}$. Subsequently, the samples were placed in another chamber with an RH of 90% at 25 °C for approximately 80 h. The final mass of the dehydrated membranes was designated as $m_{2}$. The samples’ percent moisture absorption (%*H*) was determined by gravimetry using the model presented in Equation (2).

(2) $$\%H=\left(\frac{m_{2}-m_{1}}{m_{1}}\right)\times 100$$

### 2.6. Complex Impedance Spectroscopy

The study of the ionic conductivity of the samples was carried out using a Wayne Kerr model 6420 impedance meter, where the electro-spun sample was placed between two electrodes with gold surfaces. Data were taken in the impedance cell at the frequency range of 20 Hz to 5 MHz and with a temperature range from 27 to 160 °C with a heating ramp at every 10 °C intervals. The response to the applied potential was the electrical impedance (I) of the sample. The dc conductivity was calculated from the Nyquist plots (-ImZ, ReZ). Due to the increase in the conductivity of the analyzed samples, for the heat treatment at high temperatures, the recorded spectra showed two well-defined regions: a long tail at low frequencies followed by an arc at a high-frequency limit. Consequently, the bulk resistance of the samples, *R*, was obtained from the intersection of the low-frequency tail of the spectra with a real axis. Fernandez et al., 2014 [18]. However, when the samples were subjected to lower temperatures, only the arc region was observed. Therefore, we proceeded to extrapolate the spectrum curve with the real axis. The electrical conductivity was determined by Equation (3); *L* is the thickness, and *A* is the contact area of the sample.

(3) $$\sigma =\frac{L}{R\times A}$$

where *L* is the thickness of the sample, and *A* is the cross-section of the sample.

The dependence in electrical conductivity on temperature, *T*, was analyzed using the Arrhenius model shown in Equation (4).

(4) $$\sigma =\sigma_{o}exp\left[-\frac{E_{A}}{{T\times K}_{B}}\right]$$

where *σ_o_* is the pre-exponential factor in relation to the number of charge carriers; *E_A_* is the activation energy, which represents the minimum energy required for an ion to jump from one position to another; *K_B_* is the Boltzmann constant. Based on the least-squares fit, Equation (4) was linearized with Equation (5).

(5) $$ln\sigma =ln\sigma_{o}-\frac{E_{A}}{{T\times K}_{B}}$$

## 3. Results and Discussion

### 3.1. SEM Morphology

**Figure 2:**
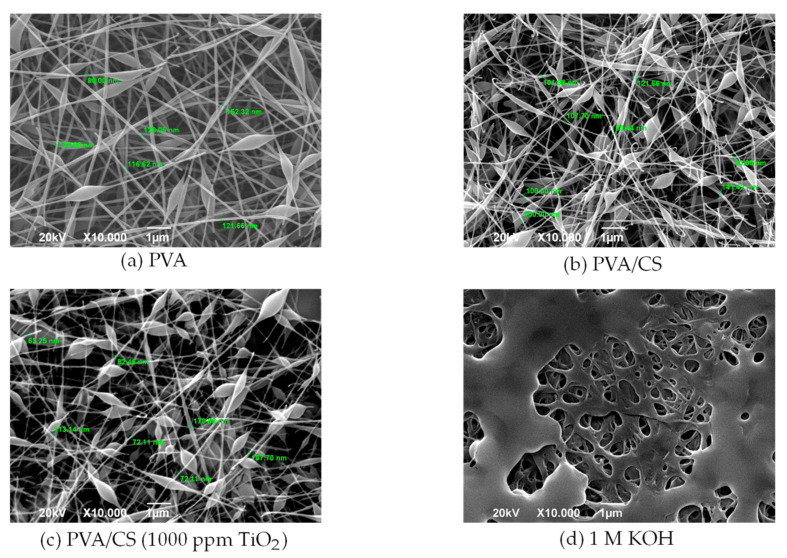
SEM micrograph of the surface section of nanofibers (**a**) PVA; (**b**) PVA/CS; (**c**) PVA/CS composite (1000 ppm TiO_2_); (**d**) 1 M KOH-doped membrane.

Figure 2 shows the SEM images corresponding to the surface morphology of the polymeric nanomembranes. In the fibrous structure of PVA, nanofibers of diameters close to 100 nm were formed. However, less uniformity was observed for the nanofibers in the binary blend. In this regard, Chun et al. (2008) [16] stated that the morphology and diameter of the nanofibers were mainly influenced by the concentration of the base solution and the proportion of the polymers in the PVA/CS binary blend. In the doping treatment (d), a porous morphology was observed due to the momentary immersion of the membrane in the KOH solution at 1 M concentration.

### 3.2. Moisture Absorption of PVA/CS-TiO_2_ (1000 to 50,000 ppm)

**Figure 3:**
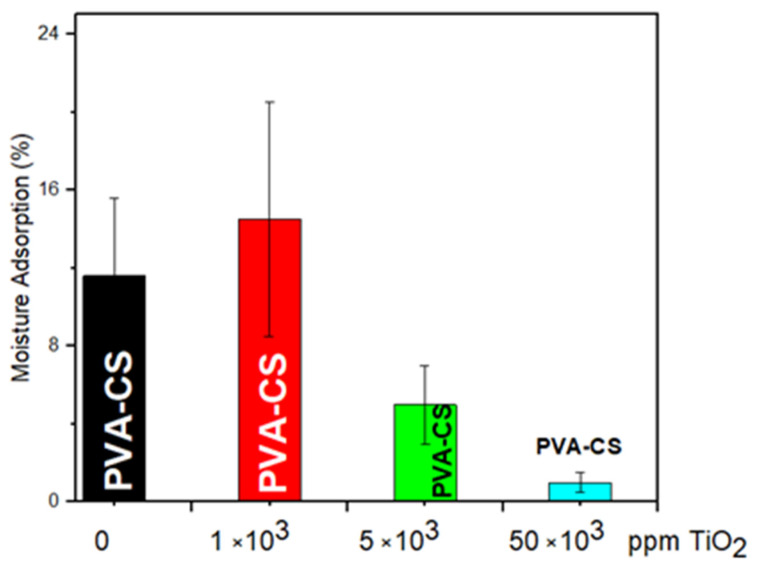
Moisture absorption for a binary blend at room temperature with different TiO_2_ contents.

Figure 3 shows the histogram of the percentage moisture absorption of the electro-spun nanocomposites (PVA/CS) as a function of a ceramic filler (TiO_2_) concentration. In the case of the binary samples without a filler, there was a 12% mass increase due to the conditioning of the samples in the humidity chamber. This mass increase could be associated with the hydrophilic nature of the polymers, which were polar groups related to water, such as hydroxyl -OH and amino -NH_2_. In addition, free water was absorbed into the nanofibers’ capillary pores and oriented within the polymer matrix by hydrogen bonds or dipoles, which were generated by interactions with the -OH groups of the polymer. By incorporating the ceramic nanoparticles into the polymeric membrane at the lowest concentration, an absorption similar to that of the unfilled membrane was obtained due to the minimal competition between the dispersed particles and the water in the pore spaces. However, for concentrations higher than 1000 ppm TiO_2_, the polymeric composite presented hydrophobic behavior, which was observed by a significant decrease in moisture absorption. It reached an equilibrium absorption value of 2% for the highest concentration of the filler in the composite. In this sense, the adequate dispersion of the ceramic filler particles could cause some defects or voids in the matrix, thus generating a free volume at the interface between the ceramic particles and the polymeric chain where the absorption and water molecule retention capacity of the polymeric composite was improved. 

In studies by Yang et al. (2011) [19], it was found that polymeric membranes (produced by casting) with high concentrations of TiO_2_ or SiO_2_ fillers presented a decrease in water absorption. The authors mentioned that this effect was possibly due to the dilution of polar groups of the polymers and the interference of the high concentrations of ceramic particles in the pore spaces that commonly occupied the water, resulting in a lower effective water absorption volume in the studied samples. In this way, Ruiz et al. (2020) [3] reported similar water absorption trends in PVA/CS binary membranes, which were formed by the casting method.

### 3.3. Thermogravimetric Analysis (TGA)

**Figure 4:**
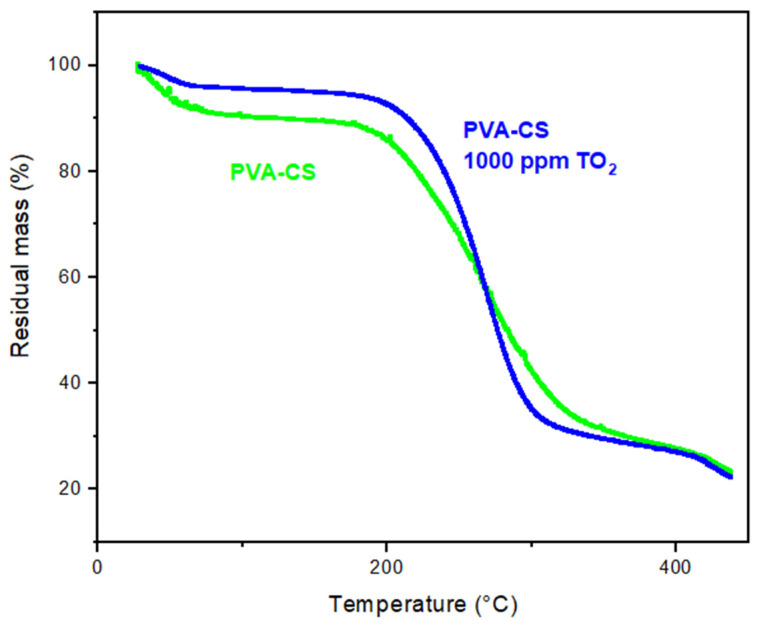
TGA curves for binary blend.

For the 1000 ppm concentration of TiO_2_ in the binary blend, significant moisture retention was observed in the composite with water loss (approximately 5% or less) for temperature increases from 30 to 200 °C (see Figure 4). That is, a synergistic effect between the binary mixture and the ceramic filler on the physical-thermal properties of the composite was evident. Similar behavior was reported by Ruiz et al. (2020) [3] and González et al. (2011) [6] in a thermal study performed on the samples (PVA-CS) and formed by solvent evaporation (casting). In another study, Vargas et al. (2000) [20] reported significant increases of up to five orders of magnitude in the ionic conductivity of PVOH/H_2_O with increasing H_3_PO_2_ doping. Mollá and Campañ (2011) [21] mentioned that effective dispersions of hygroscopic metal oxide particles, such as SiO_2_, TiO_2_, and ZrO_2_, in acidic membranes improved water retention and thermal stability. This phenomenon could be explained by the interaction between thermally stable ammonium and viable hydroxyl groups in the membranes with the ceramic filler studied, producing polymers with higher thermal stability in the composite. These results agreed with those reported by Yong et al. (2007) [12] on the usefulness of hygroscopic oxides in increasing the moisture-holding capacity of polymeric composites under low relative humidity conditions. 

### 3.4. Differential Scanning Calorimetry (DSC)

**Figure 5:**
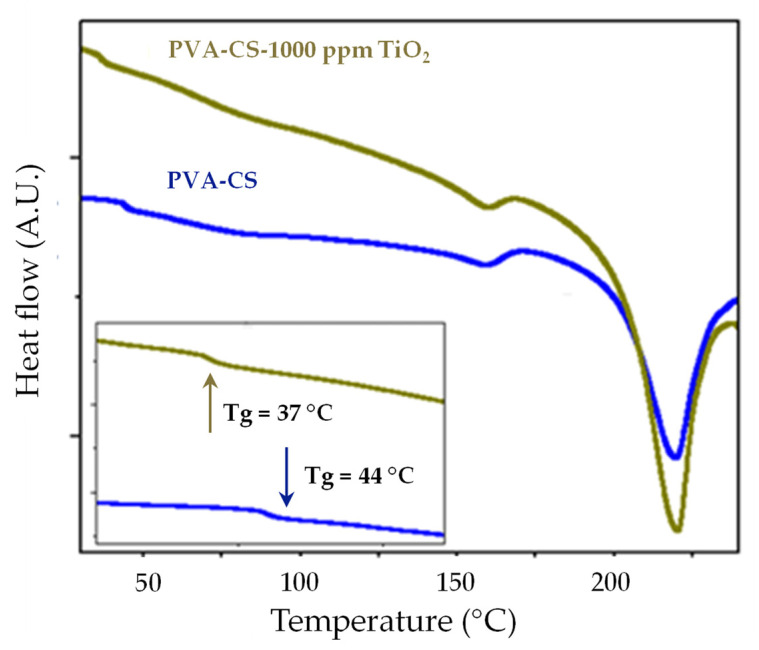
DSC curves for binary blend.

The thermal transitions for the binary mixture and the nanocomposite were studied by differential scanning calorimetry (DSC), as shown in Figure 5. This thermogram showed a characteristic step in the glass transition for the binary blend with and without nanoparticle reinforcements (1000 ppm TiO_2_) at 37 and 44 °C, respectively. The decrease in the glass transition temperature due to the effect of TiO_2_ indicated the possibility of achieving the higher ionic conductivity of the composite at room temperature. With the increasing temperature, an endothermic peak was observed for the PVA/CS binary blend at a temperature of 201 °C with an enthalpy of 43.7 J/g, which was attributable to the melting processes of the crystal lattice. This was in agreement with that investigated by Yong et al. (2007) [12], who reported a temperature (Tm) and enthalpy of fusion with values of 192.4 °C and 42.8 J/g, respectively, in thermal studies with PVA/CS electro-spun membranes. On the other hand, for the PVA/CS composite (1000 ppm TiO_2_), an endothermic peak was observed at a temperature of 203 °C and an enthalpy of 22 J/g. These results implied that the degree of crystallinity, *X_C_*, which was determined for the unfilled electro-spun sample, is higher than that estimated for the composite, with values of 39 and 20%, respectively. Therefore, the significant inhibition of crystallinity in the fibrous samples produced by the electrospinning technique was sensitive to the content of the TiO_2_ filler for the 1000 ppm concentration.

### 3.5. Complex Impedance Spectroscopy

**Figure 6:**
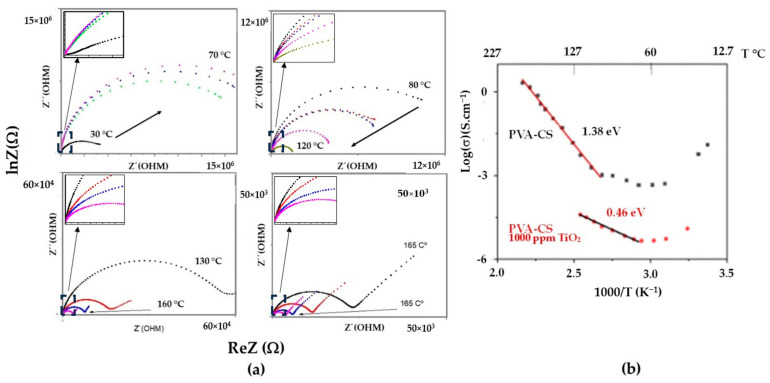
(**a**) Nyquist diagram, −Z″ vs. Z′; (**b**) Arrhenius model for binary blend.

Figure 6 shows the impedance spectrum of the PVA/CS binary membrane, which is associated with different isotherms from 30 to 190 °C. For high temperatures, an increase in conductivity was appreciated (in the spectrum of Figure 6a by the decrease in R), possibly due to the increase in the amorphous region as the binary blend approached the melting point (temperature of 201 °C, reported in the DSC analysis). The ionic conductivity of the nanomembrane was indeterminate due to the difficulty in measuring the cross-sectional thickness of the fibrous membrane, as shown in the SEM image (Figure 2). Ruiz et al. (2020) [3] reported the ionic conductivity of the composite binary, which was produced by the casting of 10^−8^ S·cm^−1^ at low moisture contents, undoped, and at room temperature. The authors reported in a test (PEMFC) an open circuit voltage for the undoped compound, which was comparable to those obtained for Nafion^®^ 117 in evaluations carried out under conditions of 90% humidity saturation. González and Betzabé [22] defined the conductivity of pure CS as ionic due to the presence of free hydroxyl groups and reported these values to be between 10^−9^ and 10^−11^ S·cm^−1^. In addition, the authors indicated that moisture absorption from the environment could increase the conductivity to 10^−4^ S·cm^−1^. Similarly, Wan et al. [23] stated that chitosan membranes were used for anion transport in aqueous solutions, achieving conductivities of 10^−2^ S·cm^−1^ with KOH-activated membranes. Additionally, solid electrolytes based on PVA/CS have been reported with conductivities on the order of 10^−2^ S·cm^−1^ by González and Vargas et al. [6], Benítez et al. [9], Quintana et al. [11], and Permana et al. [24].

Figure 6b shows the ln(σ) curves as a function of the inverse of the heating temperature on the binary blend and the nanocomposite. An average value of the membrane thickness of 15 µm was used to estimate the conductivity of the samples based on preliminary observations made with electron microscopy. This value was reliable due to the control of all the electrospinning preparation factors of polymeric nanofibers, which were indicated in the numeral 2.1. At low temperatures, a decrease in the electrical conductivity in the samples was observed in the temperature range between 30 and 70 °C. This behavior was mainly attributed to the decrease in the humidity of the membranes subjected to heat treatment. This is evident in the TGA study shown in Figure 4, where a mass loss of 5 and 10% of the binary membranes with and without nanoparticles is indicated, respectively. A linear region associated with thermal activation at high temperatures was observed. For the PVA/CS binary blend membrane, the mechanism of ionic jumps was dominant in the temperature range of 140 to 190 °C, which was influenced by the flexibility of the polymer chains due to their proximity to the polymer’s melting temperature. On the other hand, in the composite, this effect also occurred at a lower temperature range (100–150 °C), possibly due to the higher moisture retention caused by the nanoparticles dispersed in the membrane, which facilitated the ionic electromigration of the charge carriers originating from the CS. The activation energy, Ea, was calculated using the slopes and parameters of the linear regions mentioned above in Equation (5). An activation energy of 1.38 eV was obtained for the undoped binary membrane. Similarly, a lower activation energy corresponding to 0.46 eV was determined for the composite. This was due to the incorporation of PVA and the adequate dispersion of TiO_2_ in the CS matrix, which improved moisture retention and redistributed the free volume in the composite. 

**Figure 7:**
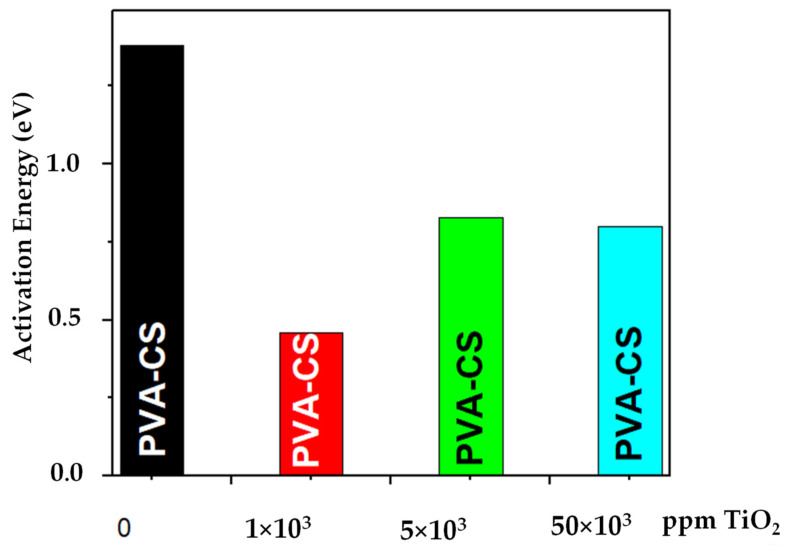
Activation energy (E_A_) for the binary blend with different TiO_2_ contents and temperature (70–160 °C).

Figure 7 presents the trend of the activation energy as a function of the filler content of the composite. Thus, the lowest value of the activation energy was presented for the TiO_2_ concentration (1000 ppm). A significant increase in the activation energy was seen for a higher nanoparticle content. In this sense, Yong et al. (2007) [9] mentioned that the ionic conductivity decreased with the increasing ceramic filler due to the dilution effect on the proton exchange groups in the original polymer matrix.

## 4. Conclusions

The moisture absorption capacity of the PVA/CS(x TiO_2_) electro-spun membranes was controlled by the incorporation of ceramic nanoparticles. For the concentration x = 1000 ppm, the hydrophilic effects of the membrane were favored, with moisture absorption up to 13%; on the other hand, at higher concentrations of the refill, the hydrophobic properties were promoted, reaching values close to 2%, perhaps due to the low competition of the hygroscopic nanofiller (concentration 1000 ppm) with the water molecules to occupy these spaces. Thermal analysis by TGA indicated in the PVA/CS composite (1000 ppm) significant moisture retention with water loss (5% or less) for temperature increments from 30 to 200 °C. This was mainly due to the synergistic interaction of the hygroscopic filler with the ionic groups of the binary membrane. In the DSC thermogram, a shift in the glass transition temperature to a lower temperature (37 °C) due to the effect of the nanoparticles (1000 ppm TiO_2_) was observed concerning the fibrous membrane without a filler (44 °C). This decrease indicated the possibility of higher ionic conductivities in the nanocomposites at room temperature. The Arrhenius model analysis for the PVA/CS composite (1000 ppm TiO_2_) inferred lower activation energy (0.46 eV) for the temperature range between 100 and 150 °C. This could be attributed both to the moisture retention capacity and to the solid plasticizing effect of the hydrophilic nanoparticles when adequately dispersed in the binary matrix. It is essential to mention that, in future work, it could be interesting to fill the intra-fibrous spaces of the electro-spun membrane with compatible material to validate the energy efficiency of the new composite as a solid polymer electrolyte in a direct methanol fuel cell.

## Data Availability

Not applicable.
